# Investigating the Inhibitory Effects of Paliperidone on RAGEs: Docking, DFT, MD Simulations, MMPBSA, MTT, Apoptosis, and Immunoblotting Studies

**DOI:** 10.3390/ijms26031060

**Published:** 2025-01-26

**Authors:** Akash Pratap Singh, Shaban Ahmad, Ahona Roy, Khalid Raza, Hemant K. Gautam

**Affiliations:** 1 Department of Botany, Maitreyi College, University of Delhi, New Delhi 110021, India; apsingh@maitreyi.du.ac.in; 2 Computational Intelligence and Bioinformatics Laboratory, Department of Computer Science, Jamia Millia Islamia, New Delhi 110025, India or shaban184343@st.jmi.ac.in (S.A.); kraza@jmi.ac.in (K.R.); 3 Infectious Disease Laboratory, Institute of Genomics and Integrative Biology (IGIB), Mathura Road, New Delhi 110025, India; ahonaroy13@gmail.com

**Keywords:** RAGEs, molecular dynamics simulation, MMPBSA, cell proliferation–apoptosis, immunoblotting

## Abstract

Chronic diseases such as diabetes and cancer are the leading causes of mortality worldwide. Receptors for Advanced Glycation End products (RAGEs) are ubiquitous factors that catalyse Advanced Glycation End products (AGEs), proteins, and lipids that become glycated from sugar ingestion. RAGEs are cell surface receptor proteins and play a broad role in mediating the effects of AGEs on cells, contributing to modifying biological macromolecules like proteins and lipids, which can cause Reactive Oxygen Species (ROS) generation, inflammation, and cancer. We targeted RAGE inhibition analysis and screening of United States Food and Drug Administration (FDA) libraries through molecular docking studies that identified the four most suitable FDA compounds: Zytiga, Paliperidone, Targretin, and Irinotecan. We compared them with the control substrate, Carboxymethyllysine, which showed good binding interaction through hydrogen bonding, hydrophobic interactions, and π-stacking at active site residues of the target protein. Following a 100 ns simulation run, the docked complex revealed that the Root Mean Square Deviation (RMSD) values of two drugs, Irinotecan (1.3 ± 0.2 nm) and Paliperidone (1.2 ± 0.3 nm), were relatively stable. Subsequently, the Molecular Mechanics Poisson–Boltzmann Surface Area (MMPBSA) determined that the Paliperidone molecule had a high negative energy of −13.49 kcal/mol, and the Absorption, Distribution, Metabolism, and Excretion (ADME) properties were in control for use in the mentioned cases. We extended this with many in vitro studies, including an immunoblotting assay, which revealed that RAGEs with High Mobility Group Box 1 (HMGB1) showed higher expression, while RAGEs with Paliperidone showed lower expressions. Furthermore, cell proliferation assay and Apoptosis assay (Annexin-V/PI staining) results revealed that Paliperidone was an effective anti-glycation and anti-apoptotic drug—however, more extensive in vivo studies are needed before its use.

## 1. Introduction

Chronic illnesses such as obesity, diabetes, and cancer are the leading causes of mortality globally [1]. The prevalence of diabetes affected 424.9 million people in 2017, with an estimated 48% increase to 628.6 million people by 2045 [2]. Concurrently, there is an increase in the prevalence of malignant neoplasms among individuals under the age of 50 [3]. Although oesophageal adenocarcinoma has a clear link to obesity, pancreatic cancer may originate with type 2 diabetes mellitus (T2DM) [4,5]. An increasing number of secondary metabolites cancers are connected to the higher incidence rate of diabetes worldwide [6,7,8,9]. The glycation process prevails in diabetes, and nonenzymatic events change glycated proteins’ molecular structure and function. Energy is produced via glycation but invariably produces reactive dicarbonyl by-products and AGEs (Advanced Glycation End products) [10]. Accumulation of AGEs in the body triggers toxic pathogenesis [11,12]. AGEs produce free radicals in oxidative stress that generate Reactive Oxygen Species (ROS) [13]. This imbalance between ROS and antioxidant defence leads to cellular oxidative stress and the accumulation of free radicals. Free radicals affect DNA, proteins, and lipids, changing biological systems and inducing malignancy.

RAGE is a multifunctional cell surface protein of the innate immune system that is estimated to play essential roles in diabetes, cancer, and other chronic inflammatory disorders [14,15,16]. Various cell types that express RAGE respond to AGE by inducing proinflammatory signalling [15]. In contrast to macrophage scavenger receptors, RAGE does not hasten the clearance of AGE, which may bind AGE and remove it from the cell environment. Instead, it causes a persistent proinflammatory signal [17]. In general, RAGE is known to bind a variety of other protein ligands, such as different S100/calgranulins, High Mobility Group Protein Box 1 (HMGB1), 2-integrin Mac-1, Carboxymethyllysine, and beta-amyloid. Upon ligand recognition, RAGE stimulates specific inflammatory responses and promotes carcinogenic events like cell activation and increased expression of cytokines and growth factors, cell migration, and the transcription factor NF-kB [18,19,20]. RAGE is a 45-kDa cell surface receptor with three immunoglobulin (Ig) domains, a single transmembrane region, and a short cytoplasmic tail at the C-terminus [21]. These domains constitute membrane-bound RAGE: a charged cytoplasmic domain that participates in intracellular signalling, an extracellular domain that identifies and binds AGE ligands, and a hydrophobic transmembrane domain [22,23].

One of the most significant methods for examining a drug’s activity using computational structure-based drug discovery is computer-aided drug discovery. Various tools evaluated the interaction between the drug library and the binding site by applying physics-based equations to determine the binding affinities of the tested compounds [24]. The effectiveness of this 1032 FDA drug is evaluated using the molecular docking technique. Further, the top four docked drugs were analysed through water-based molecular dynamic simulation, molecular mechanics with generalised Born and surface area solvation (MMGBSA), and Density Functional Theory (DFT) analysis. In vitro studies were performed to check RAGE expression analysis with the substrate (HMGB1) and with the potent inhibitor (Paliperidone), to evaluate further the Paliperidone efficacies on cell morphology and viability, and its role in apoptosis. In silico and in vitro analysess showed Paliperidone could potentially inhibit the activity of RAGE, thereby playing a crucial role in anti-ageing and cancer management and therapy. 

## 2. Results

### 2.1. Molecular Docking Studies of RAGE with FDA Compounds

**Figure 1:**
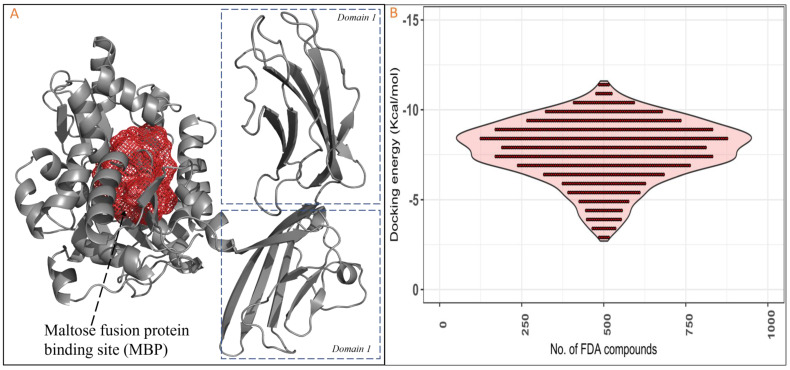
The (**A**) cartoon view of RAGEs and their active site (predicted with FTSite server), indicated by meshwork in red colour with domains highlighted differently to make it clearer, and (**B**) 1030 FDA drug binding energy with RAGEs plotted to make it clear against the number of the compounds and docking energy in kcal/mol.

**Figure 2:**
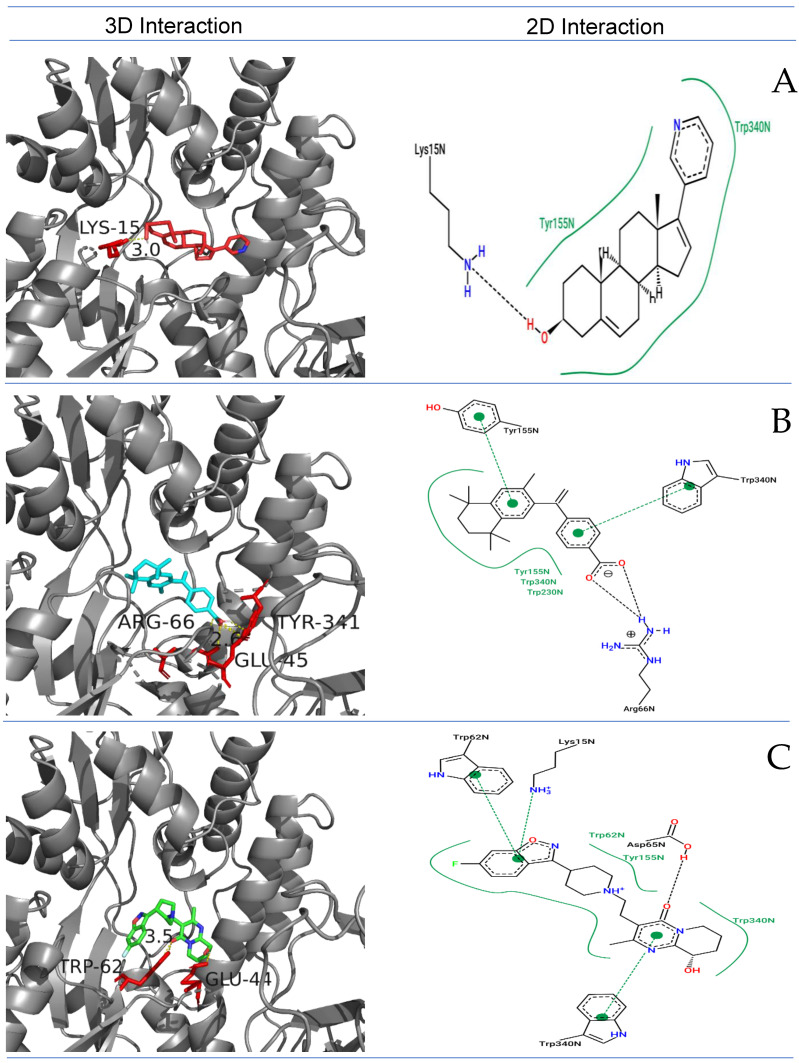

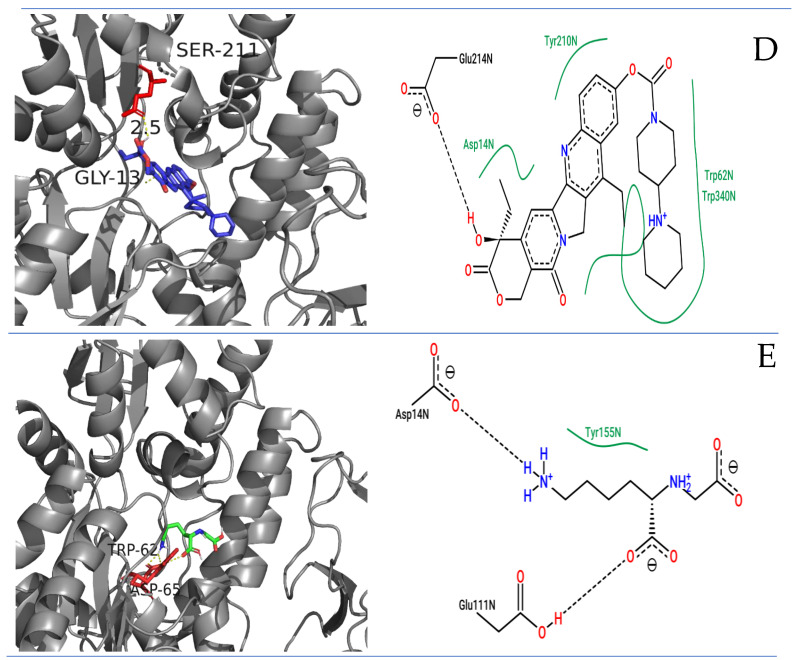
Interaction analysis of five binding dock poses in ribbon and 2D of (**A**) Zytiga (red), (**B**) Targretin (cyan), (**C**) Paliperidone (Green), (**D**) Irinotecan (blue), and (**E**) Carboxymethyllysine (green). The 2D analysis shows a black stick H-bond, and a green line indicates hydrophobic.

RAGE’s active site was predicted using the FTSite server. Demonstration of various interactions occurs between the essential active site amino acids of RAGE and the chosen FDA compounds, including hydrogen bonds and hydrophobic contacts. We docked 1030 FDA molecules to RAGE’s active site. Furthermore, based on their distinct patterns of binding interaction and energy (ΔG), we could distinguish the top four FDA medications from the 1030 FDA compounds (Figure 1). Zytiga (ZINC000003797541), Paliperidone (ZINC000004214700), Targretin (ZINC000001539579), Irinotecan (ZINC000001612996), and Carboxymethyllysine (control) were the medications examined; their respective ΔG values were −11.2, −11.2, −11.2, −11.1, and −5.0 kcal/mol, respectively (Figure 2A). In Zytiga, Lys^15^ has an H-bond with a length of ~3.0 nm, while Tyr^155^ and Trp^340^ exhibit hydrophobic interactions. Targretin demonstrated an H-bond with the Arg^66^ active site residue with ~3.6 nm bond length. Additionally, (π-π*) and nonpolar interactions were demonstrated by Tyr155 and Trp340 (Figure 2B). The Paliperidone FDA molecule showed the one-H-bond Arg^65^ with ~3.5 bond length and hydrophobic interaction; (π-π*) Tyr^68^, lys^15^, Trp^340^, Tyr^155^, Trp^340^, and Trp^62^ showed conventional nonpolar interaction (Figure 2C). The Irinotecan binding pattern demonstrated H-bond interaction between Glu^214^ and Ser^211^, with a bond length of 2.6 nm. Tyr^210^, Asp^214^, Trp^62^, and Trp^340^ indulged in hydrophobic contact (Figure 2D). Next, Carboxymethyllysine, the native substrate for RAGEs, showed two hydrogen bonds, Asp^14^ and Glu^111^, and one hydrophobic amino acid, Tyr^155^ (Figure 2E).

Among the FDA-approved compounds docked with the active site of RAGE, Paliperidone emerged as the top candidate, displaying a superior binding profile compared to the other FDA compounds and the control, Carboxymethyllysine. Paliperidone formed a crucial hydrogen bond with Arg^65^ and exhibited diverse hydrophobic interactions, including π-π* interactions with Tyr^68^, Lys^15^, and Trp^340^. Additionally, it engaged in conventional nonpolar interactions with Tyr^155^ and Trp^340^, highlighting its strong and multifaceted binding affinity. In contrast, the control compound Carboxymethyllysine showed limited interaction, with only two hydrogen bonds involving Asp^14^ and Glu^111^ and a single hydrophobic interaction with Tyr^155^—a comparatively weaker and less varied interaction pattern. Paliperidone’s binding energy (ΔG) was −11.2 kcal/mol, significantly better than the control’s (Carboxymethyllysine) −5.0 kcal/mol, indicating a stronger and more stable interaction with RAGE. The diverse interaction profiles of Paliperidone, encompassing hydrogen bonds and multiple hydrophobic contacts, suggest it can more effectively modulate RAGE activity, potentially leading to better therapeutic outcomes.

### 2.2. Molecular Dynamics Simulation Studies

**Figure 3:**
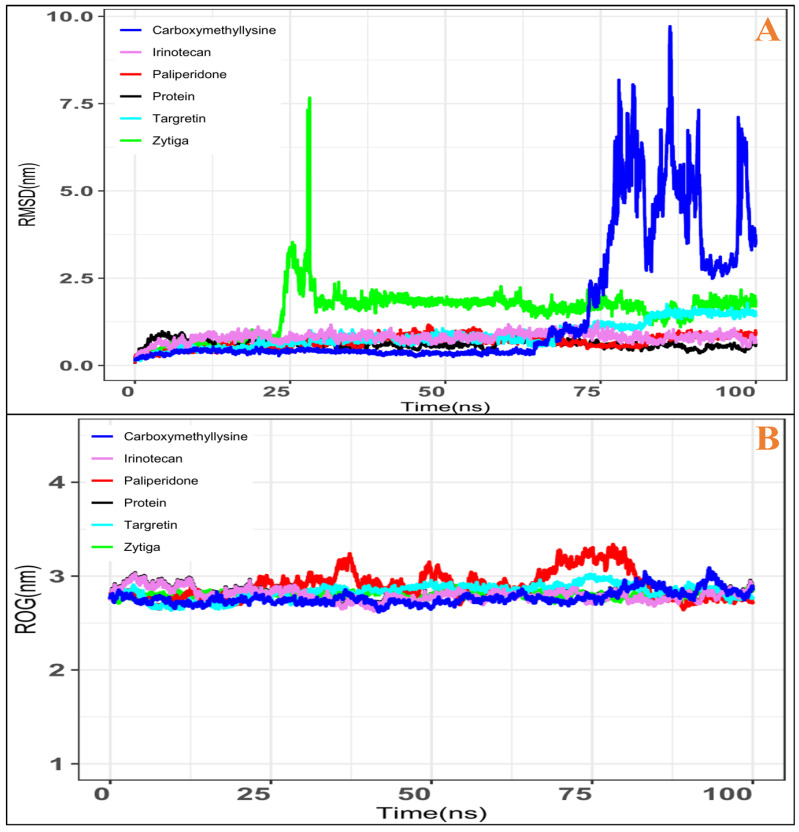
Results of 100 ns MD simulation. (**A**) RMSD trajectory for Zytiga, Paliperidone, Targretin, Irinotecan, and Carboxymethyllysine. (**B**) The gyration radius (Rg) is the same colour as the RMSD.

A water-based simulation parameter investigated the integrity of the docked complex. This experiment examined the stability of the complex in a water-filled box. Five docked compounds underwent a 100 ns production run followed by RMSD analysis (Root Mean Square Deviation). From the initial structural confirmation through the final structure confirmation, RMSD provides information on complexes (RAGE+FDA compounds). When the Zytiga trajectory was examined, it was discovered that, at 25 ns, the molecule deviated. After that, it became stable, and the average RMSD was (3.0 ± 0.4 nm). Next, the Paliperidone FDA drug molecule demonstrated better stability with no variation, and the average RMSD was (1.2 ± 0.3 nm). Further, Targretin slightly deviated at 68 ns, and the average RMSD was (1.5 ± 0.3 nm). Likewise, Irinotecan remained consistent throughout RMSD analysis, and the average RMSD was (1.3 ± 0.2 nm) (Figure 3A). Further, Carboxymethyllysine RMSDs were examined, and it was found that 0–74 ns compounds were stable; after that, from 75 ns to 100 ns, compounds were volatile, and their average RMSDs were (10 ± 1.25 nm), as shown in (Figure 3A). The Radius of Gyration (ROG) of five docked complexes was examined, offering the complexes’ compactness information. This provides information on the structure conformer deviated by the drugs, and an analysis of the 100 ns trajectory was used. Zytiga’s ROG was (~2.8 nm), Paliperidone (~3.30 nm), Targretin (~3.0 nm), Irinotecan (~2.9 nm), and Carboxymethyllysine (~2.9 nm). Further, comparative analysis showed that the (RAGE) protein’s ROG was (~2.8 nm), and that of the RAGE complex with Carboxymethyllysine was (~2.93 nm). The Paliperidone complex had a high ROG (Figure 3B), indicating good contact with the receptors, whereas Carboxymethyllysine had a low ROG, as shown in (Figure 3B).

**Figure 4:**
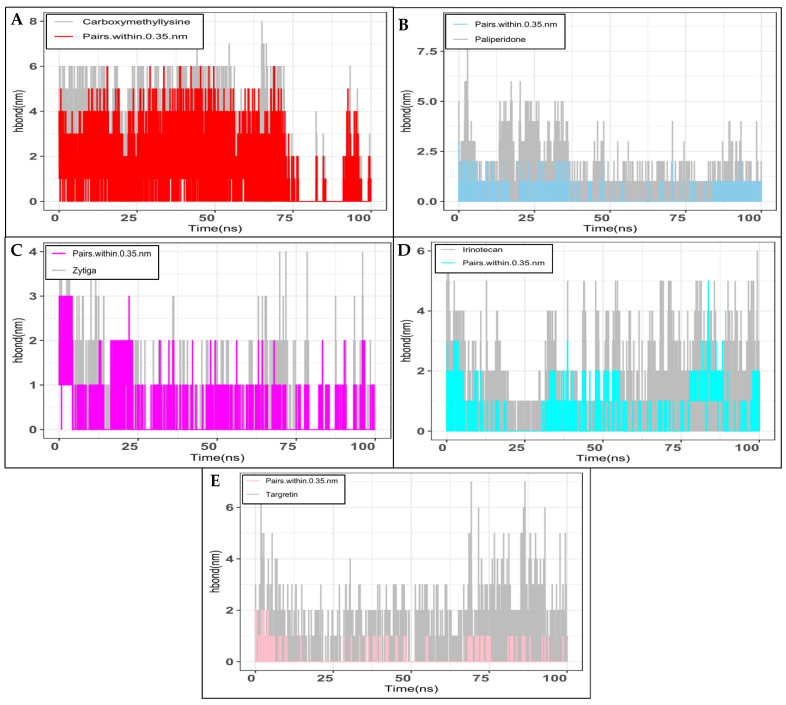
Showing the H-bond analysis (100 ns run) for the receptors for (**A**) Carboxymethyllysine, (**B**) Paliperidone, (**C**) Zytiga, (**D**) Irinotecan, and (**E**) Targretin. The colour-highlighted sticks represent the H-bond donor and acceptor atom pairs within a 0.35 nm range.

The creation of intermolecular hydrogen bonds significantly affects the stability and conformation of a protein–ligand complex. Using intermolecular hydrogen bonds may determine how strongly a ligand binds to the binding pocket of a protein. The system’s gmx H-bond module was executed during the selection of the protein and ligands. The hydrogen bonds between the protein’s 8913 atoms and the ligand’s 30 atoms were calculated using Carboxymethyllysine with RAGEs, and 793 donors and 1609 acceptor atoms were discovered. Additionally, out of the 637968 potential H-bonds, there are 2.693 on average each time period (Figure 4A). The subsequent task was to calculate the hydrogen bonds between the ligand (58 atoms) and the protein (8913 atoms) in Paliperidone using RAGEs. This revealed 792 donors and 1610 acceptors. Additionally, there is an average of 0.155 H-bonds per time frame out of the possible 637560 H-bonds (Figure 4B). In computing the hydrogen bonds between the ligand (59 atoms) and the protein (8913 atoms), calculating Zytiga with RAGEs discovered 792 donors and 1605 acceptors of atoms. Out of the 635580 potential H-bond formations, the average number of H-bonds per time frame was 0.267 (Figure 4C). Next, using RAGEs, we calculated the hydrogen bonds for Irinotecan between the protein’s 8913 atoms and the 81 ligand atoms, and discovered 792 donors and 1613 acceptor atoms. Furthermore, out of 638748 potential H-bonds, the average number of H-bonds per time frame was 0.375 (Figure 4D). Then, for Targretin, we calculated the hydrogen bonds between the protein’s 8913 atoms and the ligand’s 54 atoms using RAGEs and discovered 792 donors and 1605 acceptor atoms. Out of the 635580 potential H-bonds, the average number of H-bonds per period is 0.072 (Figure 4E).

### 2.3. MMPBSA Analysis

The binding free energy (ΔGbind) is determined by adding the molecular mechanics (MM) energy, the polar solvation energy (PB), and the nonpolar solvation energy (SA) in MM-PBSA (Molecular Mechanics Poisson–Boltzmann Surface Area) calculations. While the PB and SA components describe the solvation effects, the MM energy term represents the vdW and electrostatic interactions between the receptor and ligand molecules. A higher value of ΔG_bind in MM-PBSA calculations indicates a better binding affinity between the receptor and ligand molecules, whereas a lower or higher value denotes a weaker binding affinity. Consequently, a stronger positive value of ΔG_bind denotes a lower binding affinity between the receptor and ligand molecules. To determine the ligand–receptor affinity, the RAGEs-FDA complex was analysed, utilising the seven essential criteria of vdW, electrostatics, electrostatic contribution to the solvation free energy, electrostatic surface charge, entropy, and free energy of solvation (Gsolv). In Zytiga, the VDwaals (−21.05 kcal/mol), EEL (−1.9 kcal/mol), EPB (7.64 kcal/mol), Enpolar (−2.81 kcal/mol), Ggas (−22.95 kcal/mol), Gsolv (9.84 kcal/mol), and overall total binding energy were −12.12 kcal/mol. Next, in Paliperidone, VDwaals (−39.84 kcal/mol), EEL (4.27 kcal/mol), EPB (26.93 kcal/mol), Enpolar (−4.84 kcal/mol), Ggas (−35.58 kcal/mol), Gsolv (9.84 kcal/mol), and overall total binding energy were −12.12 kcal/mol (Table 1). Next, for Targretin, VDwaals (−25.18 kcal/mol), EEL (−2.1 kcal/mol), EPB (17.99 kcal/mol), Enpolar (−3.53 kcal/mol), Ggas (−27.27 kcal/mol), Gsolv (14.47 kcal/mol), and overall total binding energy were −12.8 kcal/mol.

Next, for Irinotecan, VDwaals (−41.25 kcal/mol), EEL (−11.45 kcal/mol), EPB (46.02 kcal/mol), Enpolar (−2.81 kcal/mol), Ggas (−4.96 kcal/mol), Gsolv (41.07 kcal/mol), and overall total binding energy were −11.52 kcal/mol. Next, for Carboxymethyllysine, VDwaals (−0.81 kcal/mol), EEL (−39.19 kcal/mol), EPB (37.13 kcal/mol), Enpolar (−0.87 kcal/mol), Ggas (−40.0 kcal/mol), Gsolv (36.27 kcal/mol), and overall total binding energy were −3.73 kcal/mol (Table 1). Paliperidone had a high total binding energy in the comparison study because VDwaals and Ggas showed substantial negative values, whereas Irinotecan exhibited VDwaals and Ggas effects but had a positive solvation energy. Electrostatic (EEL) values for Carboxymethyllysine were quite negative, whereas those for VDwaals were less negative, and those for solvation (Gsolv) energy were positive; therefore, total binding energy was less in the MMPBSA analysis.

### 2.4. DFT Computations: Paliperidone and Control Carboxymethyllysine Comparison

The Paliperidone molecule was a very stable compound after the docking, simulation, and MMPBSA analysis. Furthermore, the quantum descriptor was examined using ORCA, and the HOMO and LUMO sites of interaction pattern to RAGE were demonstrated.

**Figure 5:**
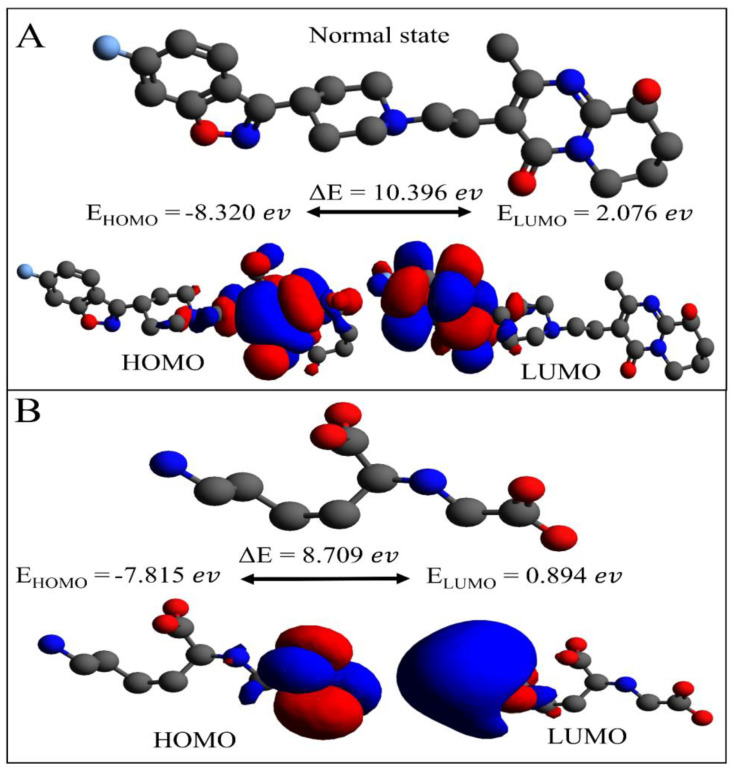
DFT analysis of (**A**) Paliperidone (selected based on MD simulation and MMPBSA analysis) compared with (**B**) Carboxymethyllysine. HOMO and LUMO sites indicate electron donating and accepting affinity.

The HOMO and LUMO may, in turn, favour a nucleophilic or an electrophilic attack on the receptor. HOMO is prone to transfer electron density, while those of the LUMO imply acceptor behaviour. These orbitals are crucial in establishing the molecule’s electrical properties and the pattern of interactions with other receptor proteins. As a result, in Figure 5, we observe the Paliperidone HOMO and LUMO orbitals, with energy HOMO −8.320 ev and LUMO state 2.076 ev. The HOMO sites are located on the doubled benzene ring, while the LUMO site is on the benzene ring integrated with the pentyl ring. The energy gap of the HOMO and LUMO molecules is E (10.396 ev), indicating that a large gap signifies the compound’s strong thermodynamic stability while a small gap indicates a simple electronic transition. The orbital energies of the Carboxymethyllysine molecule in the HOMO state are −7.815 ev and 0.894 ev, respectively. The HOMO site was found next to the oxygen atom on the right, and the LUMO site was found next to the nitrogen atom on the left. The energy gap between HOMO and LUMO revealed ΔE 8.709 ev. Comparative investigation revealed that Paliperidone had a larger energy gap, indicating that the compound was thermodynamically stable, whereas Carboxymethyllysine had a smaller energy gap difference.

### 2.5. ADME Properties of Selected Paliperidone Molecules

The ADMET analysis for the Paliperidone molecule (C23H27FN4O3) reveals several key physicochemical, lipophilic, pharmacokinetic, and drug-like properties. The molecular weight of the compound is 426.48 g/mol, with 31 heavy atoms, 15 aromatic heavy atoms, and a fraction of sp3 carbons of 0.52. The molecule has four rotatable bonds, seven hydrogen-bond acceptors, and 1 hydrogen-bond donor. The molar refractivity is 118.87, and the topological polar surface area (TPSA) is 84.39 Å^2^, indicative of good membrane permeability. The compound demonstrates high gastrointestinal absorption and is classified as soluble in water. Solubility measures include a log S (Ali) of −3.58, solubility ranging from 4.84 × 10^−2^ mg/mL to 1.13 × 10^−4^ mol/L, and moderate solubility based on SILICOS-IT predictions (−5.94 log S). It has a calculated log Kp (skin permeability) of −7.36 cm/s. In terms of lipophilicity, the log P values indicate moderate hydrophobicity, with the consensus log P being 2.96. Various log Po/w values (iLOGP: 3.86, XLOGP3: 2.17, WLOGP: 2.39, MLOGP: 3.58, and SILICOS-IT: 3.38) show consistency across models. The pharmacokinetic profile identifies Paliperidone as a P-glycoprotein (P-gp) substrate while not being a blood–brain barrier (BBB) permeant. It is an inhibitor of several cytochrome P450 enzymes, including CYP1A2, CYP2C19, CYP2C9, CYP2D6, and CYP3A4, indicating potential drug–drug interaction risks. Drug-likeness assessments show compliance with multiple filters, including Lipinski’s rule of five (no violations) and those of others such as Ghose, Veber, Egan, and Muegge. The bioavailability score is calculated at 0.55, suggesting moderate oral bioavailability. The ADMET analysis underscores Paliperidone’s suitability as a drug candidate with favourable physicochemical and pharmacokinetic properties, though its interactions with CYP enzymes warrant further investigation (Table 2).

### 2.6. Effect of Paliperidone on RAGE Expression with/Without HMGB1 Stimulation in MCF7 Cells

The effect of Paliperidone on RAGE expression was examined in MCF7 cells under conditions with and without HMGB1 stimulation. Initially, Paliperidone concentrations ranging from 0.5 µg/mL to 10 µg/mL were tested based on prior studies using small–small-molecule inhibitors in similar cellular models [25], but immunoblot experiments did not detect RAGE expression, even with HMGB1 stimulation. To overcome this limitation, the concentration range was progressively increased to 50 µg/mL and, eventually, 100 µg/mL. These higher concentrations yielded detectable RAGE expression levels, allowing us to reliably assess the dose-dependent effects of Paliperidone on RAGE inhibition and other cellular outcomes. This approach ensured that the selected concentrations were both effective and reproducible for our experimental objectives. MCF7 cells were treated with Paliperidone at 25, 50, 75, and 100 μg/mL following HMGB1 stimulation.

**Figure 6:**
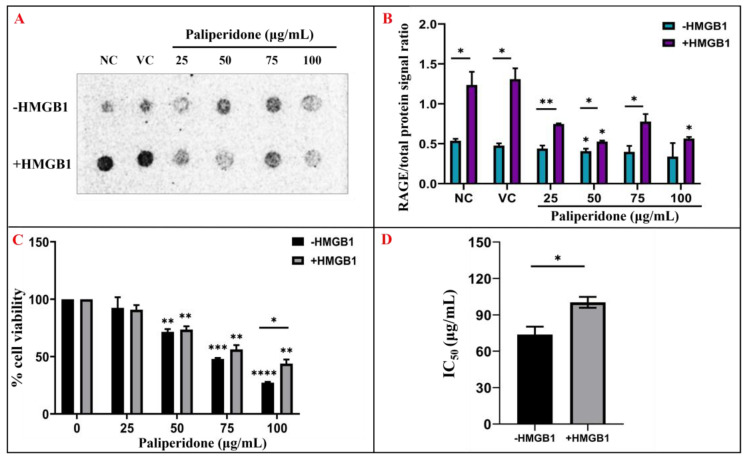
The (**A**) representative dot blot showing the effect of Paliperidone on RAGE protein expression with/without HMGB1 stimulation in MCF7 cells; NC-negative control/untreated, VC-vehicle control (DMSO). (**B**) Corresponding bar graph showing the quantitative integrated density of the bands—* *p* < 0.05, ** *p* < 0.01 versus control, n = 3, data are presented as the means +/− SE of triplicate experiments. (**C**) Representative bar graph showing the effect of Paliperidone on the proliferation of MCF7 cells with/without HMGB1 stimulation where ‘0’ signifies untreated cells; * *p* < 0.05, ** *p* < 0.01, *** *p* < 0.001, **** *p* < 0.0001 versus control, n = 3, data are presented as the means +/− SE of triplicate experiments. (**D**) Corresponding histogram denoting IC50 (conc. which causes 50% reduction in cell viability) values for Paliperidone in presence and absence of HMGB1; * *p* < 0.05, n = 3.

The results demonstrate a dose-dependent decrease in RAGE expression in HMGB1-stimulated cells as the concentration of Paliperidone increased (Figure 6). Specifically, Paliperidone at 25 μg/mL resulted in a 0.76 ratio of RAGE expression, 50 μg/mL led to a 0.6 ratio, and 100 μg/mL maintained a 0.6 ratio of RAGE expression (Figure 6). A representative dot blot (Figure 6A) analysis illustrated the effect of Paliperidone on RAGE protein expression in MCF7 cells with and without HMGB1 stimulation, including negative controls (NC, untreated) and vehicle controls (VC, DMSO). The corresponding bar graph (Figure 6B) quantified the integrated density of the bands, showing a significant decrease in RAGE expression with increasing Paliperidone concentrations. Statistical analysis indicated these changes were significant, with * *p* < 0.05 and ** *p* < 0.01 compared to the control, n = 3, and data presented as the means ± SE of triplicate experiments. Further analysis assessed the impact of Paliperidone on the proliferation of MCF7 cells with and without HMGB1 stimulation. The bar graph representation (Figure 6C) highlights a significant reduction in cell proliferation at various Paliperidone concentrations, with ‘0’ signifying untreated cells. The statistical significance of these results is marked as * *p* < 0.05, ** *p* < 0.01, *** *p* < 0.001, and **** *p* < 0.0001 versus control, n = 3, and data are presented as the means ± SE of triplicate experiments. The corresponding histogram denotes the IC50 values for Paliperidone in the presence and absence of HMGB1, demonstrating the concentration required to achieve a 50% reduction in cell viability. The statistical significance of these values is marked as * *p* < 0.05, n = 3. These results indicate that Paliperidone effectively reduces RAGE expression in a dose-dependent manner in MCF7 cells, both in the presence and absence of HMGB1 stimulation. The significant decrease in RAGE expression and cell proliferation, coupled with the calculated IC50 values, underscores the potential of Paliperidone as a therapeutic agent targeting RAGE, even outperforming the control treatments.

### 2.7. Cell Viability Effect on HMGB1-Stimulated MCF7 with Paliperidone Treatment

The impact of Paliperidone on cell viability was assessed in HMGB1-stimulated MCF7 cells. Treatment with Paliperidone at various concentrations yielded significant differences in cell viability. At 50 μg/mL, Paliperidone-treated cells exhibited 75% viability. At 75 μg/mL, cell viability decreased to 58% in the absence of HMGB1 and 61% in the presence of HMGB1. At the highest 100 μg/mL concentration, cell viability dropped to 28% without HMGB1 and 46% with HMGB1 stimulation (Figure 6C). The IC50 value, which represents the concentration required to achieve a 50% reduction in cell viability, was determined to be 73.67 μg/mL in the absence of HMGB1 and 100.3 μg/mL in the presence of HMGB1 (Figure 6D).

**Figure 7:**
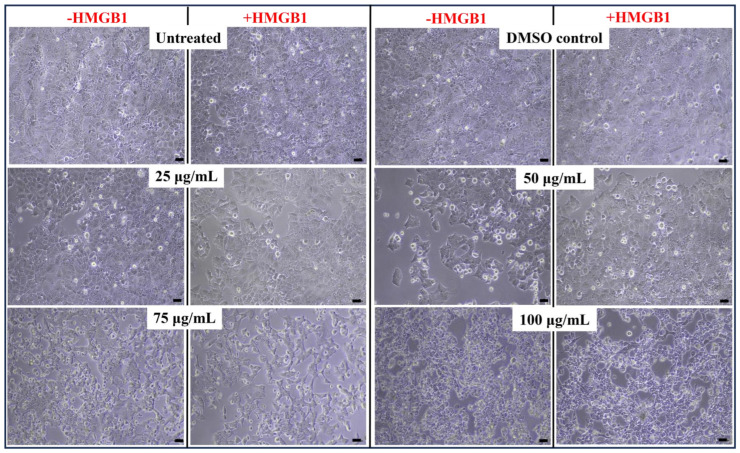
The microscopic images show the effect of Paliperidone on MCF7 cell morphology in the absence/presence of HMGB1 (scale-200 μm, 10× magnification under Nikon E200 microscope, Nikon Instrumental Inc., Melville, NY, USA).

Microscopic images provide further insights into the morphological changes induced by Paliperidone treatment in MCF7 cells (Figure 7). These images, captured at a scale of 200 μm with 10× magnification using a Nikon E200 microscope, illustrate the effects of Paliperidone on cell morphology under both HMGB1-stimulated and unstimulated conditions. The data indicate that Paliperidone exerts a dose-dependent cytotoxic effect on MCF7 cells, with significant reductions in cell viability observed at higher concentrations. The cell shape and morphology in the treated groups differed substantially from the control group. The MCF7 epithelial cells in the control group were attached to the substratum and were normally polygonal-shaped, while the ones in the treated groups appeared to be more round-shaped and became gradually fewer in number as the drug concentrations increased. Cellular debris of dead cells was also noticed in the treated groups. These findings support the potential of Paliperidone as a therapeutic agent capable of effectively reducing the viability of cancer cells, even under conditions of HMGB1 stimulation.

### 2.8. Cell Apoptosis Effect on HMGB1-Stimulated MCF7 with Paliperidone Treatment

**Figure 8:**
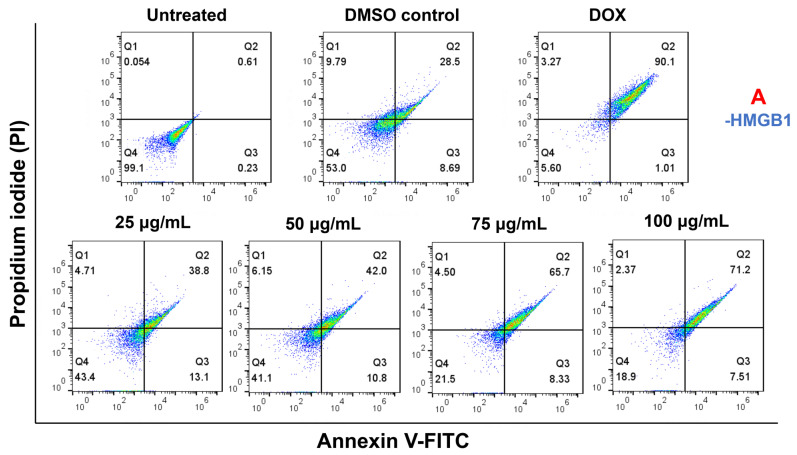

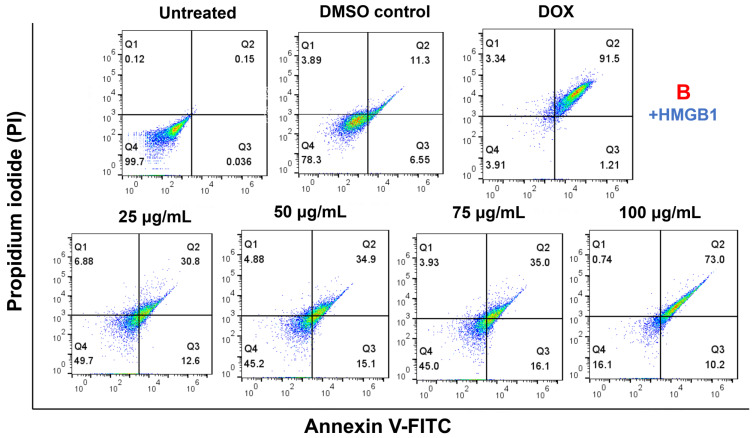

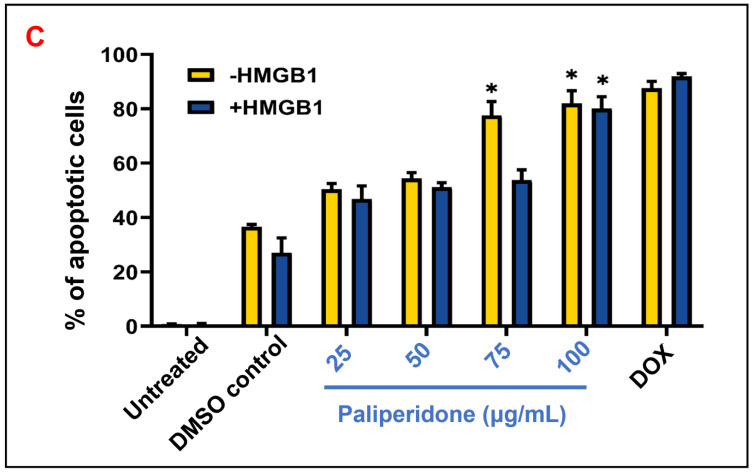
The (**A**,**B**) representative scatter plots of PI (*y*-axis) versus Annexin V (*x*-axis), showing the effect of PALI (Paliperidone) on apoptosis of MCF7 cells with/without HMGB1 stimulation; DOX-doxorubicin, a well-known pro-apoptotic compound is taken as a positive control here. (**C**) Corresponding bar graph denoting the percentage of apoptotic cells due to Paliperidone in the presence/absence of HMGB1 (* *p* < 0.05, n = 3, data are presented as the means +/− SE of triplicate experiments).

The apoptotic effects of Paliperidone on HMGB1-stimulated MCF7 cells were evaluated at various concentrations. The results show that Paliperidone induced apoptosis at 25 μg/mL in 50% of the cells without HMGB1 and 52% with HMGB1. At 50 μg/mL, the apoptosis rates were 52% without HMGB1 and 54% with HMGB1. A higher concentration of 75 μg/mL resulted in 50% apoptosis without HMGB1 and a significantly higher rate of 75% with HMGB1. At the maximum 100 μg/mL concentration, Paliperidone caused 84% apoptosis without HMGB1 and 85% with HMGB1. These findings are supported by representative scatter plots of PI (*y*-axis) versus Annexin V (*x*-axis), illustrating the apoptotic effects of Paliperidone on MCF7 cells, both in the presence and absence of HMGB1 stimulation (Figure 8A,B). Doxorubicin (DOX), a well-known pro-apoptotic compound, was used as a positive control in these experiments. The corresponding bar graph (Figure 8C) quantitatively depicts the percentage of apoptotic cells induced by Paliperidone, highlighting a significant increase in apoptosis rates, particularly at higher concentrations and with HMGB1 stimulation. Statistical analysis indicated these increases were significant (* *p* < 0.05, n = 3), with data presented as the means ± SE of triplicate experiments. The results demonstrate that Paliperidone effectively induces apoptosis in MCF7 cells dose-dependently. The presence of HMGB1 further enhances the apoptotic effect at higher concentrations, suggesting that Paliperidone could be a potent inducer of apoptosis in cancer cells, providing a strong basis for its potential therapeutic application in cancer treatment.

## 3. Materials and Methods

The methodology for identifying and validating drugs through computational and experimental approaches with each detailed methodology is as follows.

### 3.1. Retrieval of Protein and Active Site Prediction

RAGE protein’s crystal structure was first retrieved from the Protein Data Bank with PDB ID: 3O3U and a resolution of 1.50 Å and prepared to remove other heteroatoms and water from proteins by Biovia Discovery Studio. The final structure was recorded in the Protein Data Bank, Partial Charge (Q), and Atom Type T (.pdbqt) formats. The structure’s energy was then reduced using the Obabel tool [26]. Further, the FTSite server (https://ftsite.bu.edu/ (accessed on 31 March 2024)) predicts the specific ligand binding site.

### 3.2. Ligand Preparation, Selection, and ADME Analysis

A total number of 1030 FDA-approved drugs were selected from the Zinc database library (https://zinc.docking.org/ (accessed on 31 March 2024)) and saved in structure data (.SDF) format [27]. Further, the ligands were converted in .pdbqt format and minimised energy through the Obabel tool for docking studies [28,29]. Next, the best interaction molecules were analysed by the Swiss ADME server http://www.swissadme.ch (accessed on 31 March 2024).

### 3.3. Molecular Docking RAGEs with FDA Compounds

The binding conformation of the protein–ligand complex was established with molecular docking research using AutoDock v.4.2. The binding conformation would assist in the chosen ligands’ binding energies. The free binding affinities were estimated using a Lamarckian genetic algorithm, and the Root Means Square Deviation (RMSD) was investigated. Utilising polar hydrogen with fixed Kollman charges, the RAGEs were protonated. In the PDBQT received from RAGEs, partial charges, atom kinds, and torsional degrees of freedom were all mentioned [30]. However, RAGEs, ligands’ torsional bonds, and side chains were flexible. With x, y, and z coordinates of RAGE’s active site 16.656, 15.09, and 73.13, and a grid box with dimensions 50 Å × 64 Å × 52 Å was created; all ligands were docked to the residue responsible for catalytic activity. Subsequently, 10 GA (genetic algorithm) runs were conducted, with each run producing 10 postures (https://vina.scripps.edu/manual/#linux (accessed on 31 March 2024)), and each run’s best posture was recorded in a cartoon image. The Vina score of binding energy depends on the subsequent equation:

ΔG = ΔG_vdw_ + ΔG_hbond_ + ΔG_desolv_ + ΔG_elect_ + ΔG_tor_ (1)

The binding free energy (ΔG, Equation (1)), which consists of vdW, H-bond interactions, desolvation energy, electrostatic energy, and torsional free energy, was used to calculate the docking score for each posture [31]. In addition, 1030 FDA medication compounds were repurposed for docking, and comprehensive analysis was conducted from the binding posture.

### 3.4. Molecular Dynamics Simulation of RAGE and FDA-Approved Compounds

Using the Groningen Machine for Chemical Simulations (GROMACS v5.0.4) software program, a 100-nanosecond (ns) MD simulation was performed to investigate the stability of the RAGE-FDA docked complex in water [32]. The protein’s topology and force field parameters were created using the CHARMM36 all-atom force field (March 2019) program (receptor). Furthermore, the ligand force field topology file is generated through the *CHARMM General Force Field* (Cgenff) webserver (https://cgenff.umaryland.edu/ (accessed on 31 March 2024)) with default parameters. The target protein–ligand complex was then positioned in the middle of a cube with 1.0 nm-thick sides. The complex was dissolved using the TIP3-transferable intermolecular potential with three points (TIP3P) water model. The appropriate quantity of ions was added to the complexes to electro-neutralise them. By employing the 1000 steps of the steepest descent approach, bad connections and collisions in the protein were resolved. After the energy Paliperidone, all complexes underwent two equilibration phases, the first 1 ns of NVT (Number, Volume, and Temperature) equilibration and the second 1 ns of NPT (Number, Pressure, and Temperature) equilibration. Temperature coupling was used to overcome the problem of the cold solute–hot solvent by indexing the system into non-water and water components using the GROMACS gmx make ndx module [33]. A Berendsen thermostat was used to keep the system’s temperature at 310 K. Similarly, a Parrinello–Rahman barostat was used to maintain the system’s pressure. The LINCS approach was used to address the system’s long-range interaction. The MD simulations production run was kept for 100 ns; for the whole system, coordinates were stored at every 1 ns [34]. The GROMACS package’s numerous analysis (RMSD, H-bond, and Rg) modules were used to undertake structural and conformational analysis on all systems.

### 3.5. MMPBSA Calculations

These simulation trajectories were submitted to Molecular Mechanics Poisson–Boltzmann Surface Area (MMPBSA) calculations using the Amber molecular simulation tool. In receptor and ligand computation, CHARMM forcefields were used [35]. The AM1-BCC method is used to calculate the partial charges of ligands. The subsequent module is used for single-structure MM/PBSA computations, in which receptor–ligand complexes are energetically applied using the MM/PBSA method implemented in the Sander program of the Amber package [36]. For all GB computations, Onufriev and colleagues’ atomic radii are used (Amber input parameter igb = 5). For ensemble–average MM/PBSA rescoring, an MD trajectory is provided for each ligand–receptor combination. A total of 10,000 snapshots were acquired by extracting complex snapshots at 10 ps intervals from the MD trajectory. MM/PBSA approaches determine the various free energy terms of snapshot-generated complexes, receptors, and ligands [37]. The ultimate free binding energy is the mean of the snapshot’s free binding energies. In the MM/GBSA calculation, the following equations are employed to compute the binding free energy between a receptor and ligand:

ΔGbind = G_complex_ − G_receptor_ − G_ligand_ (2)

ΔGbind = ΔH − TΔS ≈ ΔE_gas_ +ΔG_sol_ − TΔS (3)

ΔEgas = ΔE_int_ + ΔE_ELE_ + ΔE_VDW_ (4)

ΔGsol = ΔE_GB_ + ΔG_Surf_ (5)

The binding free energy (ΔGbind) gives rise to several energy concepts. Since the complex, receptor, and ligand structures are derived from the same trajectory, the internal energy change (ΔEint) is nullified. Hence, the electrostatic and vdW interaction energies (ΔE_ELE_ and ΔE_VDW_, respectively) combine to provide the gas phase interaction energy (Δegas) between the receptor and the ligand. The solvation-free energy (ΔG_sol_) consists of polar and nonpolar energy components. The polar solvation energy (ΔG_GB_) is computed using the PB model. The solvent-accessible surface area (ΔG_Surf_) determines the nonpolar contribution. The dielectric constant of the solvent is set to 80, whereas the dielectric constant of the solute is set to 1. Hence, the estimated binding free energy (ΔG_bind_) is the sum of the solvation-free energy and gas-phase (entropy) interaction energy [38,39].

### 3.6. Density Functional Theory Analysis

Avogadro-1.2 and the ORCA application (latest version: 5.0.4) were used to perform geometric Paliperidone calculations using the DFT method. Avogadro’s built-in minimisation capability is applied to the structure [40,41]. The geometric Paliperidone and single-point charge calculation were produced using the ORCA output file. The optimal geometry was obtained using DFT calculations at the (Becke, three-parameter, Lee–Yang–Parr) b3lyp/made f 2SV P basis set implemented in the Orca software package. Single-point calculations on the Paliperidone structures used a large basis set and cc-pvtz (-f). Quantum calculations were performed in the ORCA software to provide thermodynamic properties and ensure that each Paliperidone obtained an energy minimisation [40,41]. ORCA provides valuable information on quantum chemical descriptors like Electronic Contribution, Nuclear Contribution, Total Dipole moment, the energy of the highest occupied molecular orbital (HOMO), and the energy of the lowest unoccupied molecular orbital (LUMO) [42]. 

### 3.7. Chemicals and Reagents

Based on the in silico investigation, Paliperidone was used for further in vitro validation studies. Paliperidone (Cat. No.: CS-0386) was purchased from ChemScene, NJ, USA. Recombinant HMGB1 protein (Cat. No.: RP00010, size: 100 µg) and anti-RAGE antibody (Cat. No.: PA5-78736) were purchased from ABclonal (Woburn, MA, USA) and Thermo Fisher Scientific, Waltham, MA, USA, respectively.

### 3.8. Cell Culture and Drug Treatment

MCF7 (human invasive breast ductal carcinoma) cells were obtained from the American Type Culture Collection (ATCC) and were grown in T-25 flasks (Thermo Fisher Scientific, MA, USA) in Dulbecco’s Modified Eagle Medium (Ref: 12800-017, HiMedia, Mumbai, India) along with 10% foetal bovine serum (Ref: RM10409-500ML, HiMedia), antibiotic antimycotic solution (Cat. No.: 15240062, Thermo Fisher Scientific, MA, USA), 3.6 g/L HEPES, and 3.7 g/L sodium bicarbonate (Sigma-Aldrich, St. Louis, MO, USA) as per manufacturer’s protocol [43]. The cells were incubated at 37 °C in a humified environment of 5% CO_2_ and 95% air. Cells were sub-cultured at 80–90% confluency in a 1:3 ratio for routine maintenance and experiments. A 0.25% trypsin–EDTA solution (Cat. No.: T4049-100ML, Sigma-Aldrich, MO, USA) was used to detach the cells from the substratum and inactivated after 5–6 min of incubation at 37 °C using equal parts of complete DMEM. The Paliperidone drug was reconstituted using DMSO to a 2 mg/mL stock, and subsequent sub-stocks and working concentrations were prepared in autoclaved MilliQ water [44]. All stocks (100 µg/mL), sub stocks, and working concentrations of recombinant HMGB1 protein were prepared in autoclaved MilliQ water.

### 3.9. Cell Proliferation Assay (MTT)

The cell proliferation assay was performed using 3-(4,5-dimethylthiazol-2-yl)-2, 5-diphenyltetrazolium Bromide (MTT). Cells were plated in 96-well cell culture flat bottom plates at a seeding density of 0.01 × 10^6^ cells/well and incubated overnight at 37 °C, 5% CO_2_ [45]. The cells were then treated with 600 ng/well of HMGB1 and, after 24 h, they were treated with different concentrations of Paliperidone drug and kept overnight in the incubator at 37 °C, 5% CO_2_. Post-treatment, 100 µL of 5 mg/mL MTT (Cat no.: M5655-100 MG, Sigma-Aldrich, MO, USA) was added to each well (final conc. of 0.5 mg/mL per well) and it was incubated in the dark for 3–4 h at 37 °C. After four hours, the MTT solution and the media were aspirated, and the formed formazan crystal precipitate was dissolved by adding 100 µL DMSO (Cat no.: 317275-100ML, Sigma-Aldrich, MO, USA) to each well. The OD was then measured at 570 nm in the Tecan Infinite F200 plate reader (Tecan Life Sciences, Zürich, Switzerland) [46]. The cell viability percentage for each drug concentration was calculated according to the following equation:

cell viability % = [(absorbance of treated cells at 570 nm)/(absorbance of untreated cells at 570 nm)] × 100%.

Each assay was performed in triplicate, and the average absorbance was calculated [47].

### 3.10. Apoptosis Assay (Annexin-V/PI Staining)

MCF-7 cells were cultured in 12-well and 24-well culture plates and incubated overnight at 37 °C, 5% CO_2_. The cells were treated with 600 ng/well of HMGB1 and, after 24 h, they were treated with different concentrations of Paliperidone drug and kept overnight in the incubator at 37 °C, 5% CO_2_ [43]. Post-treatment, after 24 h, the medium was removed; cells were harvested using trypsin and washed with ice-cold 1 × PBS twice. The pellet was resuspended in 200 μL of Annexin-binding buffer and kept on ice, followed by the addition of 5 μL Annexin V and 5 μL propidium iodide (PI) using the Annexin V-FITC Apoptosis Detection Kit (Cat. No.: ab14085, Abcam, Boston, MA, USA). A BD Accuri C6 Plus Flow Cytometer (BD Biosciences, Franklin Lakes, NJ, USA) was used to quantify the percentage of apoptotic and necrotic cells [48]. Doxorubicin hydrochloride (CAS no.: 25316-40-9, Tokyo Chemical Industry Co., Ltd., Tokyo, Japan), an extensively studied pro-apoptotic compound, was used as a positive control [49,50].

### 3.11. Immunoblotting

MCF-7 cells were plated in either 6-well or 12-well culture plates at a seeding density of 0.1–0.3 × 10^6^ cells/well and incubated overnight at 37 °C, 5% CO_2_. The cells were then treated with 600 ng/well of HMGB1 [51] and, after 24 h, they were treated with different concentrations of Paliperidone drug and kept overnight in the incubator at 37 °C, 5% CO_2_. Post-treatment, after 24 h, the medium was removed, and cells were harvested using trypsin and washed with 1 × PBS twice [52]. The total protein content was extracted using RIPA buffer (Cat. No.: R0278-50ML, MilliporeSigma, Boston, MA, USA) supplemented with 100× Protease/Phosphatase Inhibitor Cocktail (Cat. No.: PPC1010-1ML, Sigma-Aldrich, MO, USA) and centrifuged at a high speed of 14,000 rpm at 4C for 30 min. The Bradford assay estimated the protein amounts (Cat. No.: B6916-500ML, Sigma-Aldrich, MO, USA). Then, 20 µg of the total protein was loaded directly on a PVDF membrane and allowed to dry. Before sample addition, the membrane was soaked in methanol (Cat. No.: 179957-1L, Sigma-Aldrich, MO, USA) for 5 min to activate it. The PVDF membrane was blocked with 5% bovine serum albumin (Cat. No.: A3059-50G, Sigma-Aldrich, MO, USA) in TBST at room temperature for 1 h and then washed three times with TBST for 5 min each. RAGE rabbit polyclonal antibody (Thermo Fisher Scientific, MA, USA) was diluted in BSA/TBST solution (1:1000) and used to detect RAGE protein in cell lysates. The PVDF membrane was incubated with the antibody for 30 min at room temperature or overnight at 4 °C and then washed three times (5 min each) with TBST. Next, the PVDF membrane was incubated with HRP-labelled rabbit IgG secondary antibody (Cat. No.: AS014, ABclonal, MA, USA) for 1 h and washed with TBST three times for 10 min, each post-incubation. The protein bands were detected using ECL chemiluminescent reagents (Cat. No.: 32209-500ML, Thermo Fisher Scientific, MA, USA) and a ChemiDoc MP Imaging System (Bio-Rad Laboratories, Hercules, CA, USA) was used for visualisation [53].

### 3.12. Statistical Analysis

All the statistical analysis and plots were created using either GraphPad Prism (version 10.2.3, San Diego, CA, USA) or R programming language. ImageJ software (version 1.54i, Madison, WI, USA) was used to analyse the dot blot images. Data analysis for flow cytometry was carried out using FlowJo software, version 10.8.1 (BD Biosciences, Ashland, OR, USA). One-way ANOVA was used to compare the data among three or more groups, and Student’s *t*-test was used to compare two groups. In all analyses, a *p*-value less than 0.05 was considered statistically significant [54,55,56].

## 4. Discussion

Dysregulation of the signalling pathway promotes AGE accumulation which accelerates cancer development. AGEs activate ligands for several transmembrane receptors. RAGE is the most widely recognised AGE receptor. AGE–RAGE interaction activates aberrant signalling pathways and overexpressed genes [57]. The interaction promotes the activation of the NADPH-oxidase system, which results in the formation of ROS [58]. RAGE expression is elevated in pathological circumstances and chronic illnesses like cancer due to the accumulation of AGEs under stress. Consequently, ROS production, inflammation, proliferation, and angiogenesis are caused by AGE–RAGE activation [59,60]. Signal transduction ligands of RAGEs are among AGEs, and Carboxymethyllysine (CML) AGEs are one of the most common species to be discovered in human individuals [61]. Several other diseases, like an extracellular matrix (ECM) as a target of oxidative stress in the lung and the consequent formation of AGEs, are hallmarks of an in vivo ageing process that may promote fibrogenesis [62]. The daily routine life of humans may be involved in the creation of AGEs; collagen deposition, ageing, and oxidative chemicals produced by smoking, dust, or food may promote the accumulation of AGEs in lung fibrosis. To keep in mind all processes and disease mechanisms, our study demonstrates the FDA compounds that block RAGE’s activities [63,64,65]. The docking results suggest that all five selected FDA compounds, including the control (Carboxymethyllysine), were efficiently involved in the interactions with the active site amino acids of the RAGE protein through hydrogen bonding and hydrophobic interactions. Four FDA compounds showed one H-bond in 2D analysis and varied hydrophobic amino acid contribution. In contrast, Carboxymethyllysine showed two hydrogen bonds and one hydrophobic residue contribution. MD simulation studies further supported the stability of RAGEs; in RMSD, Irinotecan and Paliperidone were very stable throughout the 100 ns run, while Carboxymethyllysine, Zytiga, and Targretin deviated. ROG governs protein compactness. When a protein interacts with ligands, the compactness of the protein changes. In this, Paliperidone was high in deviation of ROG while Carboxymethyllysine, Irinotecan, Targretin, and Zytiga followed the receptor proteins. Further, the MMPBSA analysis showed significant information on VDwaals, solvation, entropy, and electrostatic interaction, and the total energies were predicted. The Paliperidone compound showed high negative energy (shown in Table 1). ADME analysis showed that Paliperidone did not violate Lipinski’s rule, and its physiological properties also indicated it as a good drug candidate. DFT analysis demonstrated the binding ability of receptors in terms of HOMO and LUMO and the energy gap between them. Paliperidone had a high energy gap (ΔE = 10.396 ev) and Carboxymethyllysine had an energy gap of (ΔE = 8.709 ev). Paliperidone showed more significant affinity to the RAGE receptor through docking, simulation, MMGBSA, and DFT.

Further, in vitro analysis was crucial for Paliperidone validation, which included various validations. In dot blot, HMGB1 was a substrate for RAGE protein, and Paliperidone was inhibiting at 25 µg/mL. Further, in MCF7 cell viability assay and apoptosis, Paliperidone was induced like an apoptotic drug. Paliperidone’s impact on RAGE expression in MCF7 cells was investigated under HMGB1-stimulated and unstimulated conditions, revealing a dose-dependent reduction in RAGE expression as Paliperidone concentration increased. The findings indicate that Paliperidone, at concentrations ranging from 25 to 100 μg/mL, significantly decreases RAGE expression levels compared to controls (Figure 6). Specifically, Paliperidone achieved a moderate reduction in RAGE expression at lower concentrations, with 25 μg/mL resulting in a 0.76 ratio and 50 μg/mL and 100 μg/mL maintaining a lower 0.6 ratio of RAGE expression (Figure 7). This reduction was consistently observed across experimental replicates, as evidenced by dot blot analyses showing decreased band densities in Paliperidone-treated cells compared to untreated and vehicle controls (NC and VC, respectively). Moreover, the quantitative assessment of band densities corroborated these findings, demonstrating statistically significant reductions in RAGE expression with increasing Paliperidone concentrations (* *p* < 0.05, ** *p* < 0.01 vs. control, n = 3). Concurrently, Paliperidone exhibited a profound inhibitory effect on MCF7 cell proliferation in a dose-dependent manner, as illustrated by bar graph representations indicating significant reductions in cell proliferation rates (* *p* < 0.05, ** *p* < 0.01, *** *p* < 0.001, **** *p* < 0.0001 vs. control, n = 3). The calculated IC50 values for Paliperidone further emphasised its potency, with values indicating the concentration required for a 50% reduction in cell viability in both HMGB1-stimulated and unstimulated conditions (* *p* < 0.05, n = 3). These comprehensive results underscore Paliperidone’s efficacy in targeting RAGE and inhibiting cellular proliferation in MCF7 cells, suggesting its potential as a therapeutic agent for conditions where RAGE-mediated pathways play a pivotal role. Paliperidone’s effect on cell viability in HMGB1-stimulated MCF7 cells was evaluated across a gradient of concentrations, revealing significant dose-dependent reductions in cell viability. At moderate concentrations, such as 50 μg/mL, Paliperidone maintained 75% cell viability, indicating its initial cytotoxic effect. As concentrations escalated to 75 μg/mL and 100 μg/mL, cell viability declined to 58% and 28% without HMGB1, respectively, and to 61% and 46% with HMGB1 stimulation (Figure 7). The determination of IC50 values further highlighted Paliperidone’s potency, necessitating concentrations of 75 μg/mL and 100 μg/mL for achieving a 50% reduction in cell viability in the absence and presence of HMGB1, respectively (Figure 7). Microscopic analysis provided visual insights into the morphological changes that Paliperidone induced, showcasing cell morphology alterations under both HMGB1-stimulated and unstimulated conditions. These observations reinforced Paliperidone’s ability to exert a cytotoxic effect on MCF7 cells, with HMGB1 stimulation mildly attenuating its impact at lower concentrations while maintaining a consistent trend of reduced viability. These findings underscore Paliperidone’s potential as a therapeutic agent capable of effectively reducing cell viability in cancer cells, even under conditions where HMGB1 stimulation might modulate cellular responses. The apoptotic effects of Paliperidone on HMGB1-stimulated MCF7 cells were thoroughly evaluated across multiple concentrations, revealing a compelling induction of apoptosis in a dose-dependent manner. From concentrations starting from 25 μg/mL to the highest tested concentration of 100 μg/mL, Paliperidone consistently promoted apoptosis, with rates escalating from 50% to 85% in the absence of HMGB1 and from 52% to 84% in the presence of HMGB1 (Figure 8A,B). The scatter plots of PI versus Annexin V provides graphical representations of Paliperidone’s apoptotic effects, further substantiating its efficacy in inducing programmed cell death. Doxorubicin was a positive control, affirming Paliperidone’s comparable or superior apoptotic activity under these experimental conditions. Quantitative analysis through bar graph presentations quantified the percentage of apoptotic cells induced by Paliperidone, revealing significant increases in apoptotic rates (* *p* < 0.05, n = 3). These findings underscore Paliperidone as a potent inducer of apoptosis in MCF7 cells, particularly evident at higher concentrations and in HMGB1 stimulation. The robust apoptotic response elicited by Paliperidone highlights its potential as a therapeutic candidate for targeting cancer cells, offering promising avenues for further exploration in cancer treatment strategies. Further, this has a limitation: it needs to be extensively validated in in vivo conditions for disease-specific problems in cancer or any other chronic disease before Paliperidone is recommended as one of the crucial drugs.

## 5. Conclusions

Conventional drug treatment has gradually undergone clinical trials and shown detrimental side effects against cancer, immunomodulation, and ageing therapies. RAGEs are a hub of various diseases, provoking growth factors through signalling. Therefore, computationally designed/identified suitable RAGE inhibitors to suppress AGE activity before clinical evaluation are an urgent and unmet need to safely manage various cancer and inflammatory diseases. In the present scenario, Paliperidone identified the promising in silico candidate compounds from FDA drug libraries through docking and simulation, and in vitro findings indicated that Paliperidone might have anti-glycation and anti-cancer properties. The comprehensive evaluation across RAGE expression, cell viability, and apoptosis assays supports Paliperidone’s multifaceted potential as a therapeutic agent targeting cancer cells, particularly in contexts where HMGB1-mediated pathways are implicated. All the studies, including computational and in vitro analysis, have supported the effectiveness of the identified drug Paliperidone against RAGEs—however, in vivo studies are recommended before its use.

## 6. Limitations of This Study and Avenues for Future Research

While this study comprehensively evaluates Paliperidone as a potential RAGE inhibitor, several limitations are associated with our approach, particularly in the in vitro experiments and the need for in vivo and toxicity studies. Since the experiments were exclusively conducted on the MCF7 breast cancer cell line, the results may not be directly translatable to breast cancer or other cancer types. Additional studies need to be conducted to evaluate Paliperidone’s anti-cancer potential and its effects on key hallmarks of cancer, such as cell migration, invasion, and angiogenesis, using assays like wound healing, transwell invasion, and tube formation. These could be performed on various cancer cell types (including different breast cancer cell lines) and non-cancerous cell lines. Testing on primary human cells could further validate cytotoxicity findings and improve translational relevance. Additionally, the in vitro environment lacks the complexity of the tumour microenvironment, including interactions with immune cells, stromal components, and an extracellular matrix, which are critical in determining drug efficacy. In future studies, incorporating tumour-immune co-culture systems, or tumour spheroids, could provide a more physiologically relevant context. While we demonstrated the induction of apoptosis and reduced RAGE expression in MCF7 cells, additional experiments can be performed using genetic knockdown studies to understand the precise pathways and mechanisms through which Paliperidone exerts its effects. Most importantly, since in vitro results do not account for Paliperidone’s systemic effects, in vivo studies are essential to understanding its behaviour, as the drug’s absorption, distribution, and metabolism affect its efficacy and safety. Given RAGE’s involvement in inflammation, this includes Paliperidone’s pharmacokinetics, bioavailability, and potential immunomodulatory roles. Animal models would help investigate its impact on nonimmune function and inflammation-related pathways. These studies would also reveal its impact on various organ systems, particularly concerning off-target effects and potential toxicity. Pharmacokinetic assessments are critical for determining appropriate dosing regimens, while organ-specific toxicity evaluations, especially for the liver and kidneys, are needed to provide a more comprehensive safety profile. By addressing these limitations, future research can provide a more holistic understanding of Paliperidone’s therapeutic potential and its application in clinical settings.

## Figures and Tables

**Table 1:** The MM-PBSA methods predicted various binding free energy values for the examined FDA drugs, represented as kcal/mol. (A) Zytiga, (B) Paliperidone, (C) Targretin, and (D) Irinotecan. VDWAALS (vdW energy), EEL (electrostatic energy), EPB (polar solvation energy), ENPOLAR (MMPBSA nonpolar solvation energy), DELTA Ggas–net gas phase energy (entropy), DELTA Gsolv (net solvation energy), and DELTA TOTAL (net system energy).

Compounds	Docking Score		MMPBSA	Analysis				
		VDWAALS	EEL	EPB	ENPOLAR	GGAS	GSOLV	TOTAL
Zytiga	−11.2	−21.05	−1.9	7.64	−2.81	−22.95	9.84	−12.12
Paliperidone	−11.2	−39.84	4.27	26.93	−4.84	−35.58	22.09	−13.49
Targretin	−11.2	−25.18	−2.1	17.99	−3.52	−27.27	14.47	−12.8
Irinotecan	−11.1	−41.25	−11.34	46.02	−4.96	−52.59	41.07	−11.52
Carboxymethyllysine	−5.0	−0.81	−39.19	37.13	−0.87	−40.0	36.27	−3.73

**Table 2:** The ADME properties of Paliperidone computed with swissADME.

Categories	Descriptors	Values	Categories	Descriptors	Values
**Physicochemical Properties**	Formula	C23H27FN4O3	**Lipophilicity**	Log S (ESOL)	−3.95
	Molecular weight	426.48 g/mol		Log Po/w (iLOGP)	3.86
	Num. heavy atoms	31		Log Po/w (XLOGP3)	2.17
	Num. arom. heavy atoms	15		Log Po/w (WLOGP)	2.8
	Fraction Csp3	0.52		Log Po/w (MLOGP)	2.39
	Num. rotatable bonds	4		Log Po/w (SILICOS-IT)	3.58
	Num. H-bond acceptors	7		Consensus Log Po/w	2.96
	Num. H-bond donors	1	**Pharmacokinetics**	BBB permeant	No
	Molar refractivity	118.87		P-gp substrate	Yes
	TPSA	84.39 Å^2^		CYP1A2 inhibitor	No
**Water Solubility**	GI absorption	High		CYP2C19 inhibitor	No
	Solubility	4.84 × 10^−2^ mg/mL; 1.13 × 10^−4^ mol/L		CYP2C9 inhibitor	No
	Class	Soluble		CYP2D6 inhibitor	Yes
	Log S (Ali)	−3.58		CYP3A4 inhibitor	Yes
	Solubility	1.13 × 10^−1^ mg/mL; 2.66 × 10^−4^ mol/L	**Drug Likeness**	Lipinski	Yes; 0 violation
	Class	Soluble		Ghose	Yes
	Log S (SILICOS-IT)	−5.94		Veber	Yes
	Solubility	4.90 × 10^−4^ mg/mL; 1.15 × 10^−6^ mol/L		Egan	Yes
	Class	Moderately soluble		Muegge	Yes
	Log Kp (skin permeation)	−7.36 cm/s		Bioavailability score	0.55

## Data Availability

Data is contained within the article and Supplementary Tables S1–S3.

## Supplementary Materials

The following supporting information can be downloaded at: https://www.mdpi.com/article/10.3390/ijms26031060/s1.
